# HIV Stigma and Discrimination: Perspectives and Personal Experiences of Healthcare Providers in Yogyakarta and Belu, Indonesia

**DOI:** 10.3389/fmed.2021.625787

**Published:** 2021-05-12

**Authors:** Nelsensius Klau Fauk, Paul Russell Ward, Karen Hawke, Lillian Mwanri

**Affiliations:** ^1^College of Medicine and Public Health, Flinders University, Adelaide, SA, Australia; ^2^Institute of Resource Governance and Social Change, Kupang, Indonesia; ^3^Infectious Disease - Aboriginal Health, South Australian Health and Medical Research Institute, Adelaide, SA, Australia

**Keywords:** HIV stigma and discrimination, perspectives, experiences, healthcare providers, Indonesia

## Abstract

Stigma and discrimination are major challenges facing People Living with HIV/AIDS (PLWHA) globally due to their HIV status. As part of a larger qualitative study in Yogyakarta and Belu, Indonesia, using in-depth interviews with 92 PLWHA (52 women, 40 men) and 20 healthcare providers, this paper describes perspectives and personal experiences of the 20 healthcare providers, relating to HIV stigma and discrimination toward PLWHA in both study settings. The healthcare providers were recruited from healthcare facilities providing HIV-related healthcare services, using a snowball sampling technique. A qualitative framework analysis was used to guide data analysis. Health stigma and discrimination framework guided the conceptualisation and discussion of the findings. The findings presented the views and perspectives of healthcare providers that HIV stigma and discrimination toward PLWHA still occurred within families, communities and healthcare settings. These were reflected in negative labelling, separation of personal belongings, avoidance, denial of treatment and rejection of PLWHA by healthcare providers, family and community members. Some healthcare providers reported that they had personally stigmatised and discriminated against PLWHA. A lack of knowledge about HIV, fear of contracting HIV, personal values, religious thoughts and sociocultural values and norms, were reported as drivers or facilitators behind this HIV-related stigma and discrimination. The findings indicate the importance of continued HIV/AIDS education for families, community members and healthcare providers, to raise awareness and to ensure that healthy and professional support systems are in place for PLWHA. The findings indicate the need to enhance improvement within the healthcare or HIV care system to adequately address the needs of PLWHA, which may facilitate their early initiation of HIV treatment and better treatment adherence and retention to increase Cluster of Differentiation 4 (CD4) count and suppress viral load. Future studies are also needed to explore the role that government and non-government institutions can play in improving health service delivery for people newly diagnosed with HIV and those living with HIV/AIDS.

## Introduction

Stigma and discrimination are major challenges facing People Living with HIV/AIDS (PLWHA) globally due to their HIV status (1, 2). Stigma is defined as a significantly discrediting attribute which serves to devalue people who possess it (3), resulting in status loss and social rejection (4). Attitudes toward the devalued attribute is often manifested as prejudice, stereotyping, and discrimination against PLWHA within families, communities and healthcare facilities (2, 3).

Stigma and discrimination against PLWHA within healthcare facilities or settings by healthcare providers have been well-documented (1, 2, 5, 6). Criticism, blaming, shouting at or throwing health records at patients' faces, and neglect or leaving patients untreated once patients' HIV status is known, are just some of the forms of discriminatory and stigmatising attitudes and behaviours reported (5, 7–10). Denial to treat, rejection and unnecessary referrals by healthcare providers due to a positive HIV status, being coerced to undergo HIV testing and abortion/sterilisation after HIV diagnosis, and loss of private health insurance are challenges PLWHA face on a regular basis (9, 10).

Previous studies have reported a range of drivers or facilitators behind these discriminatory and stigmatising attitudes and behaviours, including lack of knowledge about HIV, a lack of HIV-stigma training, HIV transmission misconceptions, and a fear of getting infected with HIV when interacting with HIV positive individuals (1, 2, 11–17). These characteristics have led to healthcare providers spending less time with PLWHA compared to other patients, and avoiding physical contact during routine medical examinations (14). Less contact with HIV patients increasingly and viciously leads to healthcare providers being less familiar with how to manage and interact with HIV patients, which in turn increases their fear of contracting HIV from patients. Negative perceptions and judgements about people infected with HIV through unprotected sex or injecting drug use (IDU), and general negative portrayals of PLWHA, are also drivers of discriminatory treatment by healthcare providers (12, 14, 18). Other factors such as healthcare providers' gender, race and religion have also been reported to play a role in discriminatory attitudes (1, 13, 14, 16, 19).

There is a paucity of research surrounding personal values, religious thoughts and beliefs and sociocultural values and norms as drivers or facilitators of HIV stigma and discrimination toward PLWHA by healthcare providers. This study aims to bridge these gaps by exploring in-depth perspectives and personal experiences of healthcare providers about HIV stigma and discrimination toward PLWHA in Yogyakarta and Belu, Indonesia. The information will be critical as the number of HIV cases in Indonesia was reported to increase significantly during the last decade, from 55,848 cases in 2010, to 377,565 cases in 2019 (20). While HIV-related stigma and discrimination are known to continue occurring throughout the country (2, 9, 19, 21, 22), an in-depth understanding about the drivers and facilitators of stigma and discrimination by healthcare providers in particular, is still very limited (2, 9, 19, 21, 22). Understanding the perspectives and experiences of healthcare providers related to HIV stigma and discrimination will be an important contribution to the current body of knowledge and useful for the improvement of HIV care systems and delivery, and to improve the health outcomes of PLWHA in Indonesia and globally.

## Methods

The consolidated criteria for reporting qualitative studies (COREQ) checklist was employed to guide the report of the methods section of this study. This checklist contains 32 required items to enhance transparency and comprehensive reporting of interviews and focus groups in qualitative studies (Supplementary Table 1) (23).

### Theoretical Framework

The Health Stigma and Discrimination Framework (HSDF) was used to conceptualise and discuss the study findings (24). This framework suggests that there are factors that drive or facilitate HIV stigma and discrimination toward PLWHA. These factors may include lack of awareness or knowledge of HIV, fear of contracting HIV infection through contact with PLWHA, fear of social ramification, blame, stereotypes, prejudice, and sociocultural norms (2, 3, 24). Drivers and facilitators determine the occurrence of stigma “marking,” through which stigma is applied to individuals or groups due to their health status or condition (e.g., HIV status) or other factors such as race, class, gender or sexual orientation (24). This framework suggests that stigma that has been applied to individuals or people manifests in discriminatory behaviours or treatments toward PLWHA by HIV negative people (2, 3, 24). It also manifests in stereotypes which reflect the beliefs about the characteristics of PLWHA that are often applied to specific individuals living with HIV, and prejudice which refers to negative emotions of uninfected people toward PLWHA and how they feel (such as disgusted, angry, and afraid) about PLWHA (2, 25, 26). Finally, the framework suggests that stigma manifestations influence PLWHA, including their access to healthcare services and adherence to HIV treatment or antiretroviral therapy (24).

### Study Setting

The study was conducted in Yogyakarta and Belu district. Yogyakarta city is a municipality in the Special Region of Yogyakarta province. It covers the area of 46 km^2^ and has a total population of 636,660 people (27). This municipality comprises 14 sub-districts and 45 villages and has a number of healthcare facilities, including two government hospitals and 18 private hospitals, 18 public health centres and nine sub-public health centres (27–29). HIV-related healthcare services in this setting were provided for HIV patients at several healthcare facilities, including four hospitals and 10 community health centres (20, 30). HIV counselling and testing, HIV information sessions, antiretroviral (ARV) medicines, cluster of differentiation 4 (CD4) and viral load tests, liver and kidney function tests and other medical tests to support HIV treatment or ARV therapy, were instances of the services provided in these healthcare facilities. Some of the services, such as HIV counselling and testing and information sessions were provided at both the healthcare facilities and through mobile system, through which the services were delivered to people within communities.

Belu district is in East Nusa Tenggara province, the Eastern part of Indonesia, and shares a border with East Timor. It covers the area of 1,284.94 km^2^, with the total population of 204,541 people including 100,922 male and 103,619 female, (31, 32). It has 12 sub-districts, 3 hospitals (one public hospital and two private hospitals), 17 community health centres, 21 sub-community health centres, 48 village maternity posts, 23 village health posts and 5 private clinics (33). It has one HIV clinic, the only healthcare facility where antiretroviral therapy (ART) is available, which is located in the public hospital. HIV-related healthcare services provided in Belu are only HIV counselling and testing and ART. The voluntary counselling and testing (VCT) are provided in the community health centres and the HIV clinic. Mobile VCT is also delivered within communities, which is preceded by HIV information sessions.

Yogyakarta and Belu are different in regards to religious perspectives, with Yogyakarta being a traditionally Muslim area with the majority of people following Javanese culture, while Belu is a traditionally Christian area where the majority of people follow Timorese culture. There is no significant differences in the number of HIV cases in the two settings, with Yogyakarta reporting 1,353 HIV cases and Belu reporting 1,200 cases (34, 35). Most PLWHA in Yogyakarta were reported to have accessed HIV care services, while only 25% in Belu accessed the services when the study was conducted (34, 35). Stigma and discrimination by healthcare providers at healthcare facilities may have been amongst negative impacts facing PLWHA and barriers for them to accessing HIV care services in these settings. However, to the best of our knowledge, there has not been qualitative inquiry which aimed to explore in-depth perspectives and experiences of healthcare providers about HIV stigma, discrimination and drivers and facilitators of stigma and discrimination toward PLWHA in the context of Indonesia, and due to feasibility, familiarity and the potential of undertaking the current study successfully, Yogyakarta and Belu were selected as the study settings.

### Study Design and Data Collection

A large-scale qualitative inquiry project using one-on-one in-depth interviews was conducted to understand HIV-risk factors and impacts on HIV positive women and men and their families in Yogyakarta and Belu, Indonesia. A qualitative design was used as it enabled the authors to explore participants' stories, understandings and interpretations about factors associated with HIV transmission among them and the experience of HIV impacts facing them and their families and their access to HIV-related healthcare services (36–38).

This paper focuses on exploring perspectives and experiences of healthcare providers on HIV stigma and discrimination and drivers and facilitators of stigma and discrimination toward PLWHA. The healthcare providers were included in the large-scale project to explore their perceptions about HIV-related health service accessibility to PLWHA across the study settings. The topic of stigma was explored as it was frequently raised in the participants' stories. Healthcare providers were recruited using the snowball sampling technique. After soliciting permission letters from the local health departments in the study settings, the study information packs were distributed to healthcare providers through healthcare facilities providing HIV-related health services. Potential participants who called and confirmed their willingness to participate in the study were asked to recommend a preferred time and place for an interview. The recruitment of the participants was an iterative process where the initial participants who had been interviewed were also asked to distribute the information packs to their colleagues or friends who might be willing to participate in this study. The inclusion criteria for the recruitment were one had to be (i) aged 18 years old or above, (ii) a healthcare professional (nurse or medical doctor) working at any healthcare facilities where HIV-related health services are available and (iii) providing HIV services for HIV patients.

One-on-one in-depth interviews were employed to collect the data from the participants. The interviews were conducted in private rooms at the healthcare facilities where the participants worked. These interview locations and times were mutually agreed upon by both the participants and the field researcher (NKF, male) who is a PhD student and had attended formal training in public health and qualitative research. The duration of the interviews with healthcare providers ranged from 35 to 58 min. Only the researcher and participant were present in the interview room and none of the participants were known to the researcher prior to this study. Interviews were conducted in Bahasa, the national language of Indonesia, and audio recorded digitally. No repeated interviews were conducted.

Recruitment of the participants and interviews ceased once the research team felt that the collected data was rich enough and data saturation had been reached. Indeed, the last few interviews provided information similar to that of previous participants, justifying our decision to cease data collection at that point. Finally, 20 healthcare providers were recruited and interviewed in the study (10 from Yogyakarta and 10 from Belu). At the end of the interviews, each participant was offered an opportunity to read and correct the information provided after the transcription, but none requested to do so.

### Data Analysis

Interview recordings were transcribed verbatim manually using a laptop and translated into English by the first author (NKF). Analysis was primarily undertaken by NKF, although team-based analysis was undertaken at regular research team meetings (NKF, PRW, KH, LM) whereby all authors undertook independent analysis and then team decisions were made about the validity of the final themes and interpretation. Cross check of the data and discussion among the research team were also conducted to maintain reliability and validity of the collected data. Transcripts were imported into NVivo 12, and data analysis was guided by a framework analysis for qualitative data by Ritchie and Spencer (39). The framework helps to manage qualitative data in a coherent and structured way, and guides the analytic process in a rigorous, transparent and valid way. Five steps of qualitative data analysis are suggested in this framework. (i) *Familiarisation* with the data or transcripts by through reading the data or transcript repeatedly, breaking down the data into small chunks of data, and providing comments or labels to the data, (ii) *identifying a thematic framework* through writing down recurrent key issues and concepts, (iii) *indexing* the *data* by creating a list of open codes through which similar or redundant codes were identified and a long list of codes was reduced to a manageable number. This was followed by closed coding to group similar codes under the same themes and sub-themes, (iv) *charting* of data by arranging appropriate thematic references in a summary of chart through which data were compared across interviews and within each interview, and (v) *mapping and interpretation of the data* where data examination and interpretations were carried out (39, 40).

## Results

### Demographic Profile of the Participants

A total of 20 HIV healthcare providers from the two study settings were interviewed for this study. Ten healthcare providers respectively were from Yogyakarta and Belu. The healthcare providers interviewed in Yogyakarta included five medical doctors, two nurses and three counsellors who were also nurses. The healthcare providers interviewed in Belu were two medical doctors and eight nurses who were also counsellors. The medical doctors, nurses and counsellors from both study settings had been involved in HIV-related health service delivery for many years ranging from 1 to 15 years. Characteristics of the healthcare providers are provided in Table 1.

**Table 1:** Sociodemographic profile of healthcare providers.

**Characteristics**	**Healthcare providers**	
	**Yogyakarta (*N* = 10)**	**Belu (*N* = 10)**
**Age**		
30–39	2	4
40–49	6	4
50–59	2	2
**Sex**		
Male	4	3
Female	6	7
**Religion**		
Islam	5	
Catholic	5	10
**Level of Education**		
Doctorate	1	
Master's degree	2	
Bachelor/Diploma	7	10
**Involvement in HIV service delivery**		
1–5 years	4	6
6–10 years		4
11–15 years	6	

### Perspectives of Healthcare Providers About HIV Stigma and Discrimination Toward PLWHA

#### Perceptions of Stigma and Discrimination in Family and Community Settings

HIV stigma and discrimination against PLWHA were reported to occur within families and communities where PLWHA lived and interacted in both Yogyakarta and Belu. Health providers across these study settings described that the fear of contracting HIV was the main factor that led to discriminatory and stigmatising attitudes and behaviours toward PLWHA by family and community members. Avoidance of physical contacts with PLWHA, labelling them as dangerous people and separation of their personal belongings from those of other family members, were some instances of HIV stigma and discrimination which reflected the fear of family and community members in these regions of the possibility of being infected with HIV. Such perceptions were drawn by the participants based on both the stories they heard from HIV patients and HIV stigma and discrimination toward PLWHA they witnessed within communities:

“*Stigma and discrimination against PLWHA still occur within families and communities even though they are not as bad as before. There are still many people who do not want to be close to PLWHA and avoid them because they are afraid of contracting HIV. They think HIV is a dangerous disease (infection) and people who are already infected with HIV are also dangerous, so they avoid PLWHA. …. There are still HIV patients who tell stories of stigma and discrimination that they experience either from their families or from those around them (neighbours and community members). I often ask them about these because I know these can prevent them from accessing the treatment. So, if we (healthcare providers) know about these then we can give them a solution. According to the report from X NGO, currently they are 38 cases of discrimination against PLWHA that they handle” (HCP7, medical doctor, Yogyakarta)*.

“*Stigma and discrimination against PLWHA are still very high here (Belu). If someone's HIV status is known to others, then the person will definitely be stigmatised and discriminated (against). He will definitely be shunned and avoided by many people for fear of being infected with HIV. What people know is that HIV is dangerous and if someone is infected then the person cannot be cured. …. It (discrimination) even happened to the ones (HIV positive people) who died. I have seen several times the discrimination that happened to them in Atambua town. People who went to see the ones (HIV positive people) who died did not want to eat or drink, did not want to stand near the corpses, some even wore masks” (HCP1, medical doctor, Belu)*.

“*People are still very scared of HIV, and stigma and discrimination against PLWHA are still high. If known that someone has HIV, then people will avoid the person. It is very difficult for people to live with HIV here because they are in a state of illness and people avoid them. …. Even family members also commit discriminatory acts. I often hear that clothes, plates, spoons, glasses and soap for PLWHA are separated from those of others because of the fear HIV transmission. There were some PLWHA here who experienced such kinds of discrimination within their families. They told me and I several times personally talked with their family members and gave them knowledge and information about HIV. Now, their family members no longer fear or discriminate against them” (HCP8, nurse and counsellor, Belu)*.

Lack of knowledge about HIV was reported as the main supporting factor for the fear of contracting HIV infection through social contact with PLWHA. The participants commented that there were still people within families and communities in in Yogyakarta and Belu who lacked knowledge or information about the means of HIV transmission. This had led to excessive fear about the possibility of contracting HIV if in contact or interacting with PLWHA, and to discriminatory and stigmatising attitudes and behaviours toward them:

“*Many people within communities do not understand about HIV. They do not know how HIV spreads from one person to another. What they do know is that HIV is dangerous and deadly. These kinds of perceptions seem to be the reasons why there are still people who are very scared of HIV, and stigma and discrimination towards PLWHA still occur within families and communities. Although, HIV stigma and discrimination have reduced a lot compared to 5 to 10 years ago but still exist” (HCP6, nurse and counsellor, Yogyakarta)*.

“*Stigma and discrimination against PLWHA still occur within families and communities because many people do not really know about the means of HIV transmission. They think that physical contact with or being close to someone who is infected with HIV, can transmit them HIV. This frightens them, so if they find out that someone is HIV positive then that person will definitely be avoided. There were HIV patients who told me and cried that their extended family members and neighbours did not want to be close to them for fear of contracting HIV.” (HCP2, medical doctor, Belu)*.

Poor participation by community members in HIV-related activities, such as information sessions or awareness raising activities and limited number of HIV-related activities carried out every year were described by the participants in Belu and seemed to be the reasons for limited dissemination of HIV information and the low level of HIV knowledge among family and community members in Belu. Meanwhile, the participants interviewed in Yogyakarta described that even though there were still family and community members who lacked knowledge about HIV, the dissemination of information about HIV among population groups and community members had significantly improved. This was due to collaboration between and different roles undertaken by the health sector and NGOs in the setting, a condition which was not reported by the participants in Belu:

“*Many community members in Belu do not know much about HIV, what they know is that HIV is dangerous and deadly. It is because not many people want to know more about HIV. Every time we carry out information socialisation about HIV within communities, only a few community members who attend” (HCP10, nurse and counsellor, Belu)*.

“*I see that many community members do not really want to know about HIV. This can be seen from the number of community members who are present when we carry out HIV information sessions, only a few. People are afraid of HIV and do not want to be tested for HIV because they are afraid of stigma and discrimination. In addition, HIV-related activities are carried out only two or three times a year, by community health centres, so if they do not attend then they will know nothing about HIV” (HCP9, nurse and counsellor, Belu)*.

“*It is true that there are still family and community members who do not know about HIV and are afraid of HIV and PLWHA. But to my opinion, information about HIV is quite widespread within groups and communities in Yogyakarta. Here, socialisation about HIV and mobile VCT are often carried out not only by community health centres and hospitals but also NGOs concerned with HIV issue. So, there is collaboration between the health sector: health department, hospitals, community health centres and NGOs. There are HIV-related activities that we implement together and there are HIV-activities that we do separately” (HCP8, medical doctor, Yogyakarta)*.

Attitudes and behaviours of HIV positive people were also described by the participants as factors that contributed to stigma and discrimination against them within communities where they lived and healthcare facilities in both Yogyakarta and Belu. Social disengagement or unwillingness to get involved in social activities or interactions with others within communities, was an instance of behaviour that raised suspicion of other community members and led to investigation of information about PLWHA. Similarly, covering the whole face to avoid being identified by other people or patients once accessing health services, was another behaviour of PLWHA that led to discriminatory behaviour toward them by other people or patients in healthcare facilities:

“*I think their (PLWHA) attitudes or behaviours also contribute to stigma and discrimination against them. There are PLWHA who do not want to socialise with other community members, do not engage in social activities within communities where they live. Of course, such behaviours make their neighbours suspicious and finally find out about their HIV status, and as the consequence, some (PLWHA) were asked to go away or move out from the communities where they lived” (HCP4, medical doctor, Yogyakarta)*.

“*There are HIV patients who cover up their entire body when accessing health services at this hospital. They wear mask, hat, jacket and cover their entire faces because they do not want to be recognised by others. I am used to seeing that other patients (HIV negative patients) do not want to sit close to them in the waiting room. Their actions make other people, other patients suspicious. Someone once asked me 'Is the person who covers his entire face an HIV patient?.” (HCP5, nurse and counsellor, Yogyakarta)*.

“*There are some HIV patients who always cover up their faces when they come to this clinic to access ARV medicines. This makes other people suspicious about them and want to find out about what disease they have. I always tell them that there is no need to cover up their faces because it will make other people suspicious of their HIV status. Strange thing (covering up face) certainly makes other people wonder” (HCP3, nurse and counsellor, Belu)*.

### Perceptions of Stigma and Discrimination in Healthcare Setting

HIV stigma and discrimination toward PLWHA were reported to also occur within healthcare facilities in Yogyakarta and Belu by healthcare providers who were not trained in the field of HIV. Participants across the study settings described that HIV patients still experienced stigma and discrimination by healthcare providers once accessing HIV-related health services in healthcare facilities. Avoidance or unwillingness to treat HIV positive patients due to the fear of contracting HIV, divulging the HIV status of PLWHA to other people and proposing offensive questions to PLWHA, were some instances of discriminatory and stigmatising attitudes and behaviours of healthcare providers toward PLWHA in both regions:

“*Many healthcare providers are still reluctant to treat HIV patients for fear of contracting HIV. Also, some healthcare providers give advice that make HIV patients feel offended, such as asking HIV patients who are gay to stop having sex with the same sex sexual partners” (HCP7, medical doctor, Yogyakarta)*.

“*Some healthcare providers propose questions that make PLWHA feel offended, such as: ‘How did you get HIV? Why do you have sex with female sex workers or same sex sexual partners?'. I know about these because there were patients who told me” (HSP1, nurse and counsellor, Yogyakarta)*,

“*Healthcare providers in this community health centre are often discriminatory against HIV patients. If they know that a patient is HIV positive, then they do not want to treat the patient, they will call me to serve the patient. They are afraid of getting HIV” (HCP9, nurse and counsellor, Belu)*.

“*There are healthcare providers in this community health centre who sometimes do not control their mouths. They divulge patients' HIV status to other people, so there are HIV patients who do not want to come to this community health centre for fear of meeting neighbours or people who know them. They are ashamed due to the assumption that other people may have already known about their HIV status through healthcare providers” (HCP4, nurse and counsellor, Belu)*.

Lack of knowledge about HIV led to the fear of contracting the infection, stigma and discrimination toward PLWHA by healthcare providers, such as doctors and nurses in both Yogyakarta and Belu. Participants interviewed across the study settings described that many healthcare providers were not trained to provide healthcare services for HIV patients and were reluctant to treat them due to the fear of contracting the infection:

“*Stigma and discrimination also often come from health workers such as doctors and nurses, especially those who work in non-HIV wards. Due to a lack of knowledge about how to treat HIV patients and fear of getting HIV, there are health workers who avoid handling HIV patients, and do not want to be close to or touch patients to carry out physical examinations” (HCP2, nurse, Yogyakarta)*.

“*There are still healthcare providers who discriminate against HIV patients, such as avoiding, refusing to examine patients physically for fear of contracting HIV. It is because many healthcare providers do not have enough knowledge about HIV (HCP8, nurse and counsellor, Belu)*.

### Barriers to Accessing Healthcare Services

HIV stigma and discrimination toward PLWHA were also reported to hinder the access to HIV-related health services among PLWHA in both Yogyakarta and Belu. Participants interviewed across the study settings described that discriminatory and stigmatising attitudes and behaviours from family members, community members and healthcare providers toward PLWHA often led to the concealment of HIV status and self-isolation, and hindered the access of PLWHA to healthcare services:

“*They (PLWHA) fear stigma and discrimination from healthcare providers who do not understand about HIV or from the community if their HIV status is discovered. This is one of the reasons why there are still HIV patients who refuse to seek treatment” (HCP1, male nurse and counsellor, Yogyakarta)*.

“*Stigma and discrimination towards PLWHA still occur, so many (PLWHA) are very careful and covering up their HIV status. That is one of the obstacles to their access to health services. This must be overcome so that PLWHA can access health services comfortably and without fear. Stigma and discrimination can come from healthcare providers: doctors and nurses in non-HIV ward, from neighbours or community members, and even from other family members” (HCP2, male nurse, Yogyakarta)*.

“*Discriminatory treatments towards PLWHA often occur and these affect their access to health services. For example, recently a patient of mine isolated himself in his house and never accessed HIV services at community health centre or VCT clinic by himself. It was because he received bad reactions neighbours and other people, so he was scared, he told me about that. He passed away last August” (HCP8, nurse and counsellor, Belu)*.

“*One of the things that makes them (PLWHA) scared of accessing HIV-related health services is stigma and discrimination from health professionals and community members. They do not come to HIV clinic because they do not want other people to know about their status. Also, the ones who get stigma and discrimination from their family members mostly do not access health services because they are not supported by their family members….” (HCP4, male nurse and counsellor, Belu)*.

### HIV Stigma and Discrimination Against PLWHA: Personal Stories by Healthcare Providers

#### Fear of HIV Transmission and Personal Values

A number of healthcare providers interviewed across the study settings (*n* = 11) reported to have committed stigma and discrimination against PLWHA. Avoidance, reluctance and unwillingness to touch or treat patients whose HIV status were known to them were some instances of discriminatory treatment toward PLWHA by these healthcare providers. Fear of HIV transmission due to limited HIV knowledge acquired prior to their involvement HIV-related health service delivery to PLWHA was described by the participants as the main driver of discriminatory and stigmatising attitudes and behaviours they had toward PLWHA:

“*I previously did not want to meet HIV patients at all. It was before I attended training on HIV and gained basic knowledge about HIV and how to treat HIV patients. So, at that time, if there was a patient who I found out that he or she was HIV positive then I avoided, I did not want to serve because I was very afraid of contracting HIV. HIV training and frequent contact with them help me to overcome the fear. After getting involved in health service delivery to them (PLHWA) for a while, I was not scared anymore and felt normal, just like I serve non-HIV patients” (HCP10, female nurse and counsellor, Belu)*.

“*Initially, I was afraid of interacting with HIV patients because I was afraid of contracting HIV. When I studied medicine, HIV was just one of the many diseases and the knowledge I got from the lecturers about HIV / AIDS was not that much. So, before I attended the special training to serve HIV patients, I still felt afraid to treat them. For example, if an HIV patient has wide wound, that makes me think twice hehehe …. I am afraid of contracting HIV, so I am kind of reluctant to touch or treat their wounds. In addition to knowledge, the experience of treating them and regular contact with them also has helped me overcame my fear. (HCP9, female medical doctor, Yogyakarta)*.

Personal values held by female participants, which were raised by male participants, were also drivers of their discriminatory and stigmatising attitudes and behaviours against PLWHA in both regions. The value of loyalty in marriage and unacceptance of cheating behaviour of PLWHA who were married as it was considered painful for their spouse, were some instances of personal values held by several participants in Yogyakarta (*n* = 4) and Belu (*n* = 4), which supported their discriminatory behaviours toward PLWHA:

“*I myself used to discriminate against HIV patients. That was because I knew what they have done is against the values I hold. For example, in marriage, the value of loyalty is very important to me, so if there is a husband who gets HIV because of having sex with other women, I do not accept it because he is cheating on his wife. As a wife, I do not accept such behaviour. That was why I previously (before attending HIV training) did not really care about male patients who were married and got HIV through sex with other women and I did not want to serve them” (HCP3, female nurse, Yogyakarta)*.

“*In my personal experience, I initially felt angry with those (HIV positive men) who got HIV because they were involved in sex with female sex workers, even though they are married. I am a wife, it certainly hurts if my husband cheats with other women. They are the ones who make mistakes, but the consequences must also be borne by their wives (HCP3, female nurse and counsellor, Belu)*.

Personal values held by these female participants also seemed to lead to personal judgement that PLWHA deserved to get the infection as the consequence of their own behaviours. Such judgement seemed to reinforce the participants' discriminatory treatment toward HIV patients:

“*I used to feel the conflict within myself every time I knew that they (PLWHA) got HIV from having sex with multiple sex partners, even though they were married. It did not feel right to me because that is unacceptable in family life or husband and wife relationship. Who would accept something like that? But as a medical doctor, I had to help them medically. This also made me initially reluctant or unwilling to serve HIV patients and made me think that they deserved to have HIV. But after attending HIV training, I started to change my perception and focus on my job as a health professional” (HCP9, female medical doctor, Yogyakarta)*.

“*In the past (before attending HIV training to be a counsellor), I did not feel empathetic or sympathetic to the male HIV patients here because I knew they got HIV through sex or cheating with FSWs (female sex workers) here or at the time they worked in Kalimantan or Malaysia. Then they passed HIV to their wives too. That is why I once thought that they deserved HIV infection. Infected with HIV was the consequence of their behaviour and it was their responsibility” (HCP10, female nurse and counsellor, Belu)*.

#### Religious Thoughts and Sociocultural Values and Norms

Religious thoughts the participants held were also facilitators of their discriminatory and stigmatising attitudes and behaviours toward PLWHA. For example, religious thoughts in Islam and Christianity that forbid the use of illicit drugs, extramarital sexual relations and consider them as sins were used by both female and male participants in Yogyakarta (*n* = 5) and Belu (*n* = 3) as parameters to judge the behaviours of PLWHA and led to unwillingness to interact with and feeling disgusted about PLWHA:

“*I admit that initially I discriminated against patients who were husbands and married who got HIV through sex with prostitutes or injecting drug use. One of the reasons was that they violated religious teachings and caused the burden not only on themselves but also on their wives. The sins and mistakes they have committed negatively affect their innocent wives and children. (HCP3, female nurse, Yogyakarta)*.

“*When I first started engaging in HIV-related health services, I felt uncomfortable and disgusted by male HIV patients who get HIV because of their engagement in free sex. They have wives but are involved in casual sex with female sex workers and get HIV. I was disgusted because I saw them attend church every Sunday but apparently their behaviours do not reflect what is taught in religion” (HCP7, female nurse and counsellor, Belu)*.

The religious thoughts the participants had also seemed to shape their negative views on PLWHA. For example, the views about PLWHA as a group of “trash people” who did not live the thoughts of their religion and the ones who were punished due to their own behaviours which were not accordant with religious thought, were some instances negative views which seemed to stem from their religious beliefs or thoughts:

“*I still remember that in the past, to me people with HIV were just a group of trash people in society. I was very strict with what is taught in my religion, and to me what they have done is wrong. I was very negative about them. All these views were washed away step by step once I started to involve in the HIV program.” (HCP4, female medical doctor, Yogyakarta)*.

“*Before I am assigned to serve HIV patients, I had a very negative view about them (PLWHA), especially those infected with HIV because of free sex. At that time, I had the view that they were punished for their own actions that were not in accordance with religious teachings, wrong and sins (HCP2, male nurse, Yogyakarta)*.

“*Before I attended training on HIV and how to serve HIV patients in 2009 or 2010, I had a very negative view about PLWHA. I thought they were punished because they behaved wrongly, were not in accordance with religious teachings and harmed their wives and children (transmitting HIV to wife and children)” (HCP2, female medical doctor, Belu)*.

The stories of a few female and male participants in Yogyakarta (*n* = 3), which were not raised by the ones in Belu, also indicated sociocultural values and norms as facilitators to their discriminatory and stigmatising attitudes and behaviours toward PLWHA. Sociocultural values and norms that do not accept sex with same sex sexual partners and considered it as a deviant and contaminated behaviour influenced their acceptance toward HIV positive patients and the way they treated and viewed PLWHA:

“*Sex with the same sex sexual partners is unacceptable socially and culturally, deviant and wrong. The norm in our social and cultural life is that a man marries a woman, not another man. It was challenging for me when I first got involved in the HIV program because to be honest I was disgusted by their sexual behaviours. I did not accept them because I thought they are infected with HIV due to their own mistakes and they deserved it. But after I attended the training and got knowledge about HIV, I began to change my views about them. ….” (HCP1, male nurse ad counsellor, Yogyakarta)*.

“*Same sex sexual behaviour is not accepted in our society and culture. It is against our social and cultural values and norms. So, initially I felt that treating them medically and teaching them to have safe sex or use condoms, are like supporting such behaviour. I quite struggled with this thought at the beginning of my involvement in HIV-related health service delivery to HIV patients. On one side I knew that they have contaminated sexual behaviours and thought that they deserved the consequences (HIV infection) but on the other side, I am a doctor who are supposed to focus on helping patients to get healthy. So, at the beginning I always had the tendency to advise them to stop and have normal relationship with girls. But due to the regular interaction with them almost every day, I also learn to understand them and focus on my task as a medical doctor” (HCP10, female medical doctor, Yogyakarta)*.

## Discussion

HIV stigma and discrimination toward PLWHA are major challenges facing PLWHA globally due to their HIV status (1, 2, 9). This paper explores in-depth perspectives and experiences of healthcare providers about HIV stigma and discrimination in Yogyakarta and Belu, Indonesia. Supporting the findings of previous studies (2, 6, 9, 41), the current study reports the perceptions of healthcare providers that HIV stigma and discrimination toward PLWHA by family and community members and healthcare providers reflected in avoidance of physical contacts with PLWHA, rejection or unwillingness to treat them, separation of their personal belongings from those of others, and negative labels, still occur within families, communities and healthcare facilities in Yogyakarta and Belu. Fear of contracting HIV through social interactions and healthcare-related contacts and lack of knowledge of HIV, as have been reported in previous findings and HSDF (1, 2, 11, 13, 17, 24, 42), were the drivers of such discriminatory and stigmatising attitudes and behaviours toward PLWHA in the study settings. The current findings suggest that lack of knowledge of the means of HIV transmission not only functions as the driver of stigma and discrimination toward PLWHA, as presented in HSDF (24), but also as the source of fear toward HIV transmission. However, as described by some healthcare providers in this study, HIV stigma and discrimination toward PLWHA in Yogyakarta seemed to have reduced. Such reduction seems to be the positive results of HIV numerous programs and activities, such as HIV information sessions for groups and communities, mobile voluntary counselling and testing, and informational support for PLWHA and family, which are carried out by healthcare providers from healthcare facilities and by several NGOs in Yogyakarta (43, 44). However, such HIV programs or activities seem to be very limited in Belu, with the local health department in the district as the only sector responsible for HIV issue.

Supporting the constructs of HSDF (24), the current study confirms that personal attitudes and behaviours of PLWHA in both study settings also contribute to stigma and discrimination against them. For example, covering face on accessing healthcare services and avoiding social engagement or interaction with neighbours and other community members to conceal self-identity and HIV positive status actually acted as drivers of HIV stigma and discrimination against PLWHA as these led to suspicion and investigation of information about them by other patients or community members. In societies that emphasise collectivism, such as in Indonesia (2, 45), covering face and avoiding social interactions or social disengagement, which are not common behaviours, will definitely raise curiosity and suspicion of other people to find out information about PLWHA, which could increase the likelihood of them being stigmatised and discriminated.

The study findings also suggest facilitators to HIV stigma and discrimination toward PLWHA by healthcare providers which have not been explored in-depth in previous studies (1, 14, 24). For example, personal values of female healthcare provider participants across the study settings, such as husband and wife loyalty and unacceptability of cheating behaviours in marriage, were indicated as the facilitators to their discriminatory and stigmatising attitudes and behaviours toward PLWHA. Such personal values lead to the participants' projection of what happened in the marriage of PLWHA (e.g., husbands had sex with other women or were unfaithful and contracted HV, which were considered unacceptable and painful) to their own situation or marriage, which reinforced their negative attitudes and behaviours toward PLWHA. The current findings also suggest that female healthcare providers had more discriminative and stigmatising attitudes toward PLWHA compared to male healthcare provider participants. These are in line with results of previous studies (14, 46), reporting that female doctors and ward staff had significantly more negative feelings and stigmatising attitudes toward PLWHA compared to their male colleagues.

Religious thoughts in Islam and Catholicism that forbid the use of illicit drugs, extramarital sexual relations and consider them as sins were also facilitators of HIV stigma and discrimination by healthcare providers. The use of such thoughts as the parameters to judge the behaviours of PLWHA leads to the healthcare providers' unwillingness to serve, interact with and feeling disgusted about HIV patients. These support the findings on a previous study (47), reporting that the inclusion of personal religious beliefs in health delivery to PLWHA led to the clash between personal religious values and professional expectations. However, previous studies (47, 48) have also reported personal religious beliefs as motivators or facilitators for some healthcare providers' health service delivery to PLWHA, an aspect which was not diagnosed among the participants in the current study. Although religion as one of the predictors to HIV stigma and discrimination by healthcare providers toward PLWHA has been reported in the findings of several previous studies (1, 13, 14, 16, 19), but the mechanisms through which religion contributes to HIV stigma and discrimination, as explored in the current study, were not explored in-depth in those studies. Sociocultural values and norms that do not accept sex with same sex partners and consider it as a deviant and contaminated behaviour also influenced the participants' acceptance toward HIV positive patients, treatment and perceptions about PLWHA or facilitated HIV stigma and discrimination toward PLWHA by the participants. The findings of previous studies in Indonesia have reported that general perceptions about sex with same sex partner as deviant and contaminated behaviours prevented men who have sex with men and transgender people to openly talk about their health status and to seek healthcare services (49–52).

Personal values, religious thoughts and sociocultural values and norms held by healthcare providers in the current study were also facilitators of their negative judgements toward PLWHA as “trash people” and people who deserved HIV infection as a punishment or consequence of their behaviours (e.g., extramarital sex or sex with multiple sex partners or with same sex partners). It should be acknowledged that stigma and discrimination toward PLWHA by health professionals who participated in this study were committed prior to or at the beginning of their involvement in HIV-related health service delivery to PLWHA, a condition where they had not attended HIV training and were not equipped with proper knowledge on HIV and how to serve HIV patients. Knowledge of HIV and health service delivery to PLWHA acquired through HIV training they attended and the experience of treating HIV patients, helped them overcome the fear of contracting HIV from patients and supported their non-discriminatory and stigmatising attitudes and behaviours toward PLWHA. These support the findings of previous studies (41, 42, 53), which have reported that exposure to HIV-related knowledge or having in-depth HIV knowledge, attending training of stigma and discrimination, gaining more experience in treating HIV patients and frequent contact with them, are associated with low level of stigmatising attitudes or negative predictors of stigma and discrimination among health professionals. Supporting the constructs of HSDF and the findings of previous studies (1, 2, 24, 42), the current study also suggests that HIV stigma and discrimination are hindering factors for the access to healthcare services among PLWHA and also lead to concealment of HIV status and self-isolation of PLWHA.

## Study Limitations and Strengths

The study cannot be complete without pointing out its limitations. The inclusion criteria for recruitment which required healthcare providers who were involved in HIV healthcare service delivery to PLWHA and the use of snowball sampling technique might have been limitations as these might have resulted in the recruitment of participants from the same networks and the neglection of the perspectives and experiences of healthcare providers who were not directly involved in health service delivery to PLWHA. However, the strengths of the study were that the study purpose was clearly identified, and the use of qualitative design helped the researchers explore in-depth the views, perspectives and experiences of the participants about the topic being studied. The use of a framework analysis to guide this qualitative data analysis was also a strength as it helped the management of these qualitative data in a coherent and structured way, and enhanced transparency, rigour and validity of the analytic process. Besides, to our knowledge, this is the first qualitative inquiry to focus on exploring perspectives and experiences of healthcare providers about HIV stigma and discrimination and drivers and facilitators of stigma and discrimination toward PLWHA by healthcare providers in the context of Indonesia. The current findings are useful for the improvement of healthcare system and delivery that address the needs of PLWHA in the study settings and in Indonesia as whole and other similar settings globally.

## Conclusions

The current study reports on perspectives and experiences of healthcare providers about HIV stigma and discrimination or drivers and facilitators of stigma and discrimination toward PLWHA in Yogyakarta and Belu, Indonesia. It reports that HIV stigma and discrimination toward PLWHA still occurred within families, communities and healthcare settings in both settings. These were reflected in negative labelling by others, separation of their personal belongings from those other family members, avoidance of physical contacts by community members, and rejection or unwillingness to treat and the spread of HIV status of PLWHA by healthcare providers. Lack of knowledge of HIV, fear of contracting HIV from PLWHA, personal values, religious thoughts and sociocultural values and norms, were reported as the drivers or facilitators of HIV stigma and discrimination toward PLWHA. These led to the concealment of HIV status and self-isolation and hindered the access of PLWHA to healthcare services. The findings indicate the importance of HIV/AIDS education for family and community members, and healthcare providers to enhance their knowledge and awareness of HIV/AIDS, and to accept PLWHA. The findings also indicate the need for the improvement of healthcare system and delivery to address the needs of PLWHA. Future studies that explore what can be done by government and non-government institutions to improve health service delivery to PLWHA are recommended.

## Data Availability Statement

The datasets presented in this article are not readily available because the dataset is a set of interview transcripts - we cannot (due to restrictions set by the human research ethics committee) provide these transcripts to other researchers. Requests to access the datasets should be directed to paul.ward@flinders.edu.au.

## Ethics Statement

Ethics approvals for this study were obtained from Social and Behavioural Research Ethics Committee, Flinders University, Australia (No. 8286), and the Health Research Ethics Committee, Duta Wacana Christian University, Yogyakarta, Indonesia (No. 1005/C.16/FK/2019). Prior to the interviews, each participant was informed about the purpose of the study and that the study had obtained ethical approvals. Study participants were advised about the voluntary nature of their participation and that they had the right to withdraw their participation at any time, without consequence, if they felt uncomfortable with the questions being asked. They were also advised that the interview would take ~45–90 min and would be recorded using a digital recorder. They were assured that the data or information that they provided during the interview was confidential and unidentifiable, as each participant was assigned with a specific study identification letters and number. This was to prevent the possibility of linking back the data or information to any individual in the future. Each participant received reimbursement of IDR 100,000 (±USD 7) for transport and their time. Before commencing the interviews, each participant signed the informed consent form and returned it to the researcher.

## Author Contributions

NF was involved in the conceptualisation of the study, development of the methodology, data collection and analysis, and in drafting the manuscript, revising it critically for important intellectual content and integrating the comments of the reviewers. PW, KH, and LM were involved in the conceptualisation of the study, development of the methodology, supervision, and in revising the manuscript critically for important intellectual content. All authors approved the final manuscript.

## Conflict of Interest

The authors declare that the research was conducted in the absence of any commercial or financial relationships that could be construed as a potential conflict of interest.
